# Analysis of the association of *ANO3/MUC15*, *COL4A4*, *RRBP1*, and *KLK1* polymorphisms with COPD susceptibility in the Kashi population

**DOI:** 10.1186/s12890-022-01975-3

**Published:** 2022-05-05

**Authors:** Lifeng Tang, Xuemei Zhong, Hui Gong, Maimaitiaili Tuerxun, Tao Ma, Jie Ren, Chengxin Xie, Aifang Zheng, Zulipikaer Abudureheman, Ayiguzali Abudukadeer, Paierda Aini, Subinuer Yilamujiang, Li Li

**Affiliations:** 1 Department of Respiratory and Critical Care Medicine, First People’s Hospital of Kashi, Kashi, 844000 Xinjiang People’s Republic of China; 2 Clinical Research Center of Infectious Diseases (Pulmonary Tuberculosis), First People’s Hospital of Kashi, Kashi, 844000 Xinjiang People’s Republic of China

**Keywords:** Chronic obstructive pulmonary disease, *ANO3/MUC15*, *COL4A4*, *KLK1*, *RRBP1*, Genetic polymorphism, Smoking status

## Abstract

**Objective:**

Chronic obstructive pulmonary disease (COPD) is a complex, multifactorial, polygenic disease. The rate of occurrence of COPD in the Kashi population (Uyghur) is significantly higher than that observed nationwide. The identification of COPD-related genes in the Chinese Uyghur population could provide useful insights that could help us understand this phenomenon. Our previous whole-exome sequencing study of three Uyghur families with COPD demonstrated that 72 mutations in 55 genes might be associated with COPD; these included rs15783G > A in the anoctamin 3 (*ANO3*) gene/mucin 15 (*MUC15*) gene, rs1800517G > A in the collagen type IV alpha 4 chain (*COL4A4*) gene, rs11960G > A in the ribosome binding protein 1 (*RRBP1*) gene, and rs5516C > G in the kallikrein 1 (*KLK1*) gene. This case–control study aimed to further validate the association of the four mutations with COPD in the Chinese Uyghur population.

**Methods:**

Sanger sequencing was used for the genotyping of four polymorphisms (*ANO3/MUC15* rs15783, *COL4A4* rs1800517, *RRBP1* rs11960, and *KLK1* rs5516) in 541 unrelated Uyghur COPD patients and 534 Uyghur healthy controls. We then conducted stratified analyses based on the smoking status and airflow limitation severity, to explore the correlation between selected gene polymorphisms and COPD.

**Results:**

*ANO3/MUC15* rs15783 and *KLK1* rs5516 polymorphisms could significantly reduce COPD risk (*p* < 0.05), but *COL4A4* rs1800517 and *RRBP1* rs11960 polymorphisms were not correlated with COPD in the entire population. In a stratified analysis of smoking status, non-smokers with the *ANO3/MUC15* rs15783G/G genotype (OR = 0.63, *p* = 0.032) or *COL4A4* rs1800517 allele G (OR = 0.80, *p* = 0.023) had a reduced risk of COPD. Smokers with the *RRBP1* rs11960A/G genotype had a lower risk of COPD (OR = 0.41, *p* = 0.025). The *KLK1* rs5516G > C polymorphism was associated with a decreased risk of COPD (OR < 1, *p* < 0.05), irrespective of the smoking status of individuals. No significant association with COPD severity was observed in individuals with these four polymorphisms (*p* > 0.05).

**Conclusion:**

We identified four previously unreported mutations (*ANO3/MUC15* rs15783, *COL4A4* rs1800517, *RRBP1* rs11960, and *KLK1* rs5516) that might decrease the COPD risk in individuals with different smoking statuses in the Chinese Uyghur population. Our findings provide new light for the genetic risk factors associated with the occurrence of COPD.

**Supplementary Information:**

The online version contains supplementary material available at 10.1186/s12890-022-01975-3.

## Introduction

Chronic obstructive pulmonary disease (COPD) is characterized by persistent respiratory symptoms and airflow limitations [1], which are correlated with higher morbidity and mortality worldwide [2]. Cigarette smoking (CS) is the leading environmental risk factor for COPD; yet, even among heavy smokers, less than 50% develop COPD during their lifetime [3]. Epidemiologic studies have consistently shown that people who have never smoked might develop chronic airflow limitations [4]. In addition, during our previous epidemiological investigation in Kashi (Xinjiang, China), three three-generation families had presented with COPD [5]. The mechanisms responsible for the occurrence of these phenomena are unclear, but could depend at least partially on the genetic makeup of individuals. The epidemiolog ical investigation also showed that the rate of occurrence of COPD in Chinese Uighurs whose age was above 40 years in Kashi was 17.01% [5] and was significantly higher than that observed nationwide [6]. The identification of COPD-related genes in the Chinese Uyghur population could provide useful insights that could help us understand this phenomenon.

**Fig. 1:**
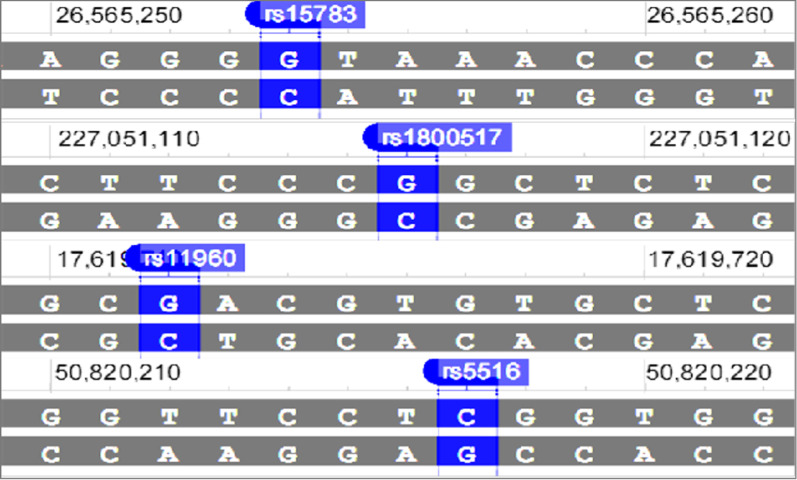
The physical location of *MUC15* rs15783, *COL4A4* rs1800517, *RRBP1* rs11960, and *KLK1* rs5516 (Genes, NCBI Homo sapiens Annotation Release)

Protein-coding genes constitute only ~ 1% of the human genome but harbor 85% of all mutations and significantly influence the occurrence of disease-related traits; thus, whole-exome sequencing (WES) can be used to obtain relevant insights into diverse human diseases [7]. Therefore, we performed WES on eight people with COPD and one healthy person from three Uyghur families with COPD in Kashi to screen for the susceptibility genes and polymorphisms related to COPD [8]. WES facilitated the identification of 72 single nucleotide variants (SNVs) of 55 genes, including g.26565254G > A (rs15783G > A, NC_000011.10) in the anoctamin 3 (*ANO3*) gene/mucin 15 (*MUC15*) gene, g.227051116G > A (rs1800517G > A, NC_000002.12) in the collagen type IV alpha 4 chain (*COL4A4*) gene, g.17619712G > A (rs11960G > A, NC_000020.11) in the ribosome binding protein 1 (*RRBP1*) gene, and g.50820217C > G (rs5516C > G, NC_000019.10) in the kallikrein 1 (*KLK1*) gene [8] (Fig. 1 and Additional files 1, 2, 3 and 4). Notably, the WES study evaluated only one healthy control as a reference; the screened 72 SNVs might include SNVs found in healthy people. Hence, it was necessary to verify the relationship between these 72 SNVs and COPD in a case–control study. Moreover, although scholars have found that several gene polymorphisms might be related to COPD susceptibility [9, 10], the correlation between polymorphisms of the four genes (*ANO3/MUC15* rs15783, *COL4A4* rs1800517, *RRBP1* rs11960, and *KLK1* rs5516) and COPD risk is still poorly understood. Therefore, a case–control study of 1075 individuals (541 COPD patients and 534 healthy subjects) recruited from the same population was conducted, to evaluate the correlation between four selected SNVs and COPD risk. Furthermore, the correlations between the four selected SNVs and both clinical and functional parameters were explored. We found that the *ANO3/MUC15*, *KLK1*, *COL4A4*, and *RRBP1* polymorphisms were closely associated with a decreased risk of COPD in individuals with different smoking statuses. The four SNVs were not found to be correlated with COPD severity after a stratified analysis based on airflow limitation severity. Our findings would provide further insights into the mechanism of occurrence of COPD.

## Materials and methods

### Experimental subjects

Individuals included 541 Uyghur unrelated COPD patients and 534 Uyghur healthy controls were consecutively recruited from the First people’s Hospital of Kashi during the period from 2018 to 2019. The age of all the participants was > 40 years. Spirometry (Cosmed, Rome, Italy) was performed for all subjects. COPD was diagnosed in accordance with the diagnostic criteria outlined by the global initiative for chronic obstructive lung disease (GOLD), which specified that the post-bronchodilator forced expiratory volume in 1 s (FEV1)/forced vital capacity (FVC) should be < 70% [1]. Baseline characteristics and classical COPD risk factors were recorded. None of the subjects were diagnosed with a history of atopy and an α1-antitrypsin deficiency. Additional inclusion and exclusion criteria for the participants were described by Gong et al. [11].

Informed written consent was obtained from all subjects. This study was carried out with the approval (Approval details: Kuai Shen Yan No. 70) of the Ethics Committee of the First People's Hospital of Kashi. Five milliliters of peripheral blood samples were obtained from each of the 1075 participants for DNA extraction and transferred to BD Vacutainer ® EDTA-K2 blood collection tubes for DNA extraction.

### Target gene sequencing

Genomic DNA samples were extracted from the peripheral blood using the Genomic DNA purification Kit (TIANGEN BIOTECH (BEIJING) CO.,LTD, China), in accordance with the manufacturer's instructions. The NanoDrop 2000 (Thermo Fisher Scientific, Waltham, MA, USA) was used to measure the concentration and quality of extracted DNA. The four SNVs (*ANO3/MUC15* rs15783, *COL4A4* rs1800517, *RRBP1* rs11960, and *KLK1* rs5516) were genotyped via Sanger sequencing using a custom-by-design 48-Plex SNPscan Kit (Center for Genetic & Genomic Analysis, Genesky Biotechnologies Inc., Shanghai, China) [8]. The ABI3730XL (Applied Biosystems, USA) sequenator and GeneMapper 4.1 (Applied Biosystems, USA) were adopted to analyze the polymorphisms.

### Statistical analysis

SPSS 18.0 statistical software (SPSS Inc., Chicago, IL, U.S.A.) was used for statistical analysis. Quantitative data were presented as means ± standard deviations (SD) for normally distributed values or medians plus interquartile ranges for non-normally distributed data. Categorical variables were described in terms of the count (%). Pearson's chi-squared test was used to compare the differences in gender, smoking status, smoking index (SI), wood consumption, and coal consumption. The differences in age and body mass index (BMI) between different groups were assessed using an independent sample t-test, after quantile–quantile (QQ) plots demonstrated that these data showed an approximately normal distribution. FEV_1_%, FEV_1_/FVC, and annual household income values that did not conform to the normal distribution were calculated using the Mann–Whitney U test. Fisher's exact test was used to assess the variations in all the SNVs frequencies by assessing the Hardy–Weinberg equilibrium (HWE). Odds ratios (ORs) and 95% confidence intervals (CIs) were calculated to determine the association between the selected SNVs and COPD risk using logistic regression analysis, with adjustments for age, gender, and BMI. Using PLINK software (version 2.0), the effects attributable to single nucleotide polymorphisms (SNPs) were fitted under five models of inheritance, i.e., the additive, codominant, dominant, recessive, and allele models. The stratified model was applied for subgroup analysis after taking the smoking status and pulmonary function of participants into consideration. We used *p* < 0.05 as the cut-off value for determining statistical significance.

## Results

### Characteristics of the study population

The clinical characteristics and spirometry data of 1075 participants in the case–control study have been provided in Table 1. A total of 541 COPD patients (280 male and 261 female) and 534 controls (234 male and 300 female) were included in the study. There was no significant difference between COPD cases and controls in terms of wood consumption, coal consumption, and annual household income (*p* > 0.05). The differences between the two groups with regard to age, gender, BMI, smoking status, smoking index, FEV1%, and FEV1/FVC were statistically significant (*p* < 0.05). Within the COPD population, 150, 292, 82, and 17 patients had mild disease (FEV1 ≥ 80% predicted), moderate disease (50% ≤ FEV1 < 80% predicted), severe disease (30% ≤ FEV1 < 50% predicted), and very severe disease (FEV1 < 30% predicted), respectively.

**Table 1:** General characteristics of COPD patients and healthy subjects

Variables	Case (n = 541)	Control (n = 534)	*p*
Age, years (mean ± SD)	61.11 ± 12.26	54.86 ± 10.73	< 0.001
Gender (male/female), n	280/261	234/300	0.009
BMI (kg/m^2^) (mean ± SD)	23.56 ± 4.22	25.55 ± 4.17	< 0.001
Annual household income (CNY, yuan) (median, range)	16,939 (10,294–23,442)	15,298 (9762–23,057)	0.197
Smoking status (never/former/current), n	417/23/101	465/17/52	< 0.001
Smoking index^a^(< 1/1 ≤ SI ≤ 400/400 < SI ≤ 800/SI > 800), n	417/ 98/16/10	465 /52/11/6	< 0.001
Coal consumption (yes/no), n	513/28	516/18	0.144
Wood consumption (yes/no), n	519/22	500/34	0.09
Time-to-first respiratory symptoms^b^, years (≤ 5/5 < Time ≤ 10/ > 10), n	196/277/68	–	–
GOLD grade (1/2/3/4), n	150/292/82/17	–	–
Domiciliary oxygen therapy (nasal catheter/NIV/no), n	243/107/191	–	–
*Pulmonary drugs (inhalant), n*			
Monotherapy (SABA/SAMA/LAMA)	11/5/79	-	-
Combination therapy (LABA + LAMA/ICS + LABA/ICS + LAMA + LABA)	85/138/30	-	-
Other (no/unavailable)	169/24	–	–
*Pulmonary drugs (oral), n*			
Theophylline	174	–	–
LTRA	197	–	–
Other (no/unavailable)	139/36	–	–
Lung function (median, range)			
FEV_1_%	69.00 (53.92–82.00)	86.00 (74.00–98.00)	< 0.001
FEV_1_/FVC	0.62 (0.55–0.66)	0.80 (0.75–0.86)	< 0.001

*FEV1* Forced expiratory volume in 1 s, *FVC* Forced vital capacity, *BMI* Body mass index, *NIV* Noninvasive ventilation, *SABA* Short-acting beta2-agonist, *SAMA* Short-acting muscarinic antagonist, *LABA* Long-acting beta2-agonist, *LAMA* Long-acting muscarinic antagonist, *ICS* Inhaled corticosteroids, *LTRA* Leukotriene receptor antagonist, *GOLD* Global initiative for chronic obstructive lung disease

^a^Smoking index = number × year

^b^Respiratory symptoms, including cough, sputum production, and dyspnea

### Genotype analyses of COPD risk

Four SNVs (*ANO3/MUC15* rs15783, *COL4A4* rs1800517, *RRBP1* rs11960, and *KLK1* rs5516) of target genes were successfully genotyped in the case–control study. The call success rate was more than 99%. Genotype distributions of the above SNVs were in accordance with Hardy–Weinberg equilibrium predictions (*p* > 0.05) (Table 2).

**Table 2:** Basic information and allele frequencies among all SNVs

Gene	SNV ID	Chromosome Position	Common Allele	Minor Allele	MAF		HWE
					Cases	Controls	
*ANO3/MUC15*	rs15783	Chr11: 26,586,801	A	G	0.382	0.410	0.339
*COL4A4*	rs1800517	Chr2: 227,915,832	A	G	0.473	0.501	0.803
*RRBP1*	rs11960	Chr20: 17,600,357	A	G	0.437	0.425	0.160
*KLK1*	rs5516	Chr19: 51,323,473	G	C	0.266	0.301	0.114

*SNV* Single nucleotide variant, *MAF* Minor allele frequency, *HWE* Hardy–Weinberg equilibrium

To identify whether the polymorphisms of these genes were related to COPD susceptibility, we analyzed five genetic models (additive, codominant, dominant, recessive, and allele) that were adjusted for age, gender, and BMI (Table 3). The results showed that the *ANO3/MUC15* rs15783G > A and *KLK1* rs5516C > G polymorphisms were correlated with a decreased risk of COPD (*p* < 0.05). Individuals with the *ANO3/MUC15* rs15783 G/G genotype had a reduced risk of COPD, as shown by the results obtained with the codominant model (OR = 0.67, *p* = 0.038) and recessive model (OR = 0.67, *p* = 0.021). The *KLK1* rs5516 C/C genotype was associated with a decreased risk of COPD under the codominant model (OR = 0.62, *p* = 0.042) and recessive model (OR = 0.62, *p* = 0.039). No significant association was observed for other SNVs.

**Table 3:** Analysis of genotypes of *ANO3/MUC15* rs15783, *COL4A4* rs1800517, *RRBP1* rs11960, and *KLK1* rs5516

Genotype frequency, n (%)								
SNV ID	Model	Genotype	Case N (%)	Control N (532)	OR(95%CI)^a^	*p*^a^	OR(95%CI)^b^	*p*^b^
*ANO3/MUC15* rs15783	Codominant	A/A	203	196	1		1	
		A/G	262	237	1.07(0.82–1.39)	0.628	1.01(0.77–1.34)	0.932
		G/G	76	100	0.74(0.52–1.05)	0.089	0.67(0.46–0.98)	**0.038**
	Dominant	A/A	203	196	1		1	
		G/G, A/G	338	337	0.97(0.76–1.24)	0.799	0.91(0.70–1.18)	0.476
	Recessive	A/A, A/G	465	433	1		1	
		G/G	76	100	0.71(0.51–0.98)	**0.037**	0.67(0.47–0.94)	**0.021**
	Additive	–	–	–	0.90(0.75–1.06)	0.202	0.86(0.71–1.02)	0.087
	Allele	A	668	629	1		1	
		G	414	437	0.89(0.75–1.06)	0.196	0.85(0.71–1.02)	0.082
*COL4A4* rs1800517	Codominant	A/A	157	137	1		1	
		A/G	255	258	0.86(0.65–1.15)	0.313	0.81(0.60–1.10)	0.173
		G/G	128	138	0.81(0.58–1.13)	0.212	0.75(0.53–1.07)	0.114
	Dominant	A/A	157	137	1		1	
		G/G, A/G	383	396	0.84(0.65–1.10)	0.216	0.79(0.60–1.05)	0.104
	Recessive	A/A, A/G	412	395	1		1	
		G/G	128	138	0.89(0.68–1.17)	0.407	0.86(0.64–1.16)	0.319
	Additive	–	–	–	0.90(0.76–1.06)	0.208	0.87(0.73–1.03)	0.109
	Allele	A	569	532	1		1	
		G	511	534	0.90(0.76–1.06)	0.198	0.86(0.72–1.03)	0.102
*RRBP1* rs11960	Codominant	A/A	170	179	1		1	
		A/G	267	254	1.11(0.84–1.45)	0.463	1.04(0.79–1.39)	0.789
		G/G	102	99	1.09(0.77–1.54)	0.646	1.00(0.69–1.44)	0.992
	Dominant	A/A	170	179	1		1	
		G/G, A/G	369	353	1.10(0.85–1.42)	0.462	1.03(0.79–1.35)	0.841
	Recessive	A/A, A/G	437	433	1		1	
		G/G	102	99	1.02(0.75–1.39)	0.895	0.98(0.71–1.35)	0.877
	Additive	–	–	–	1.05(0.89–1.25)	0.573	1.00(0.84–1.20)	0.962
	Allele	A	607	612	1		1	
		G	471	452	1.05(0.89–1.25)	0.572	1.00(0.84–1.20)	0.962
*KLK1* rs5516	Codominant	G/G	289	269	1		1	
		G/C	210	202	0.97(0.75–1.25)	0.800	0.98(0.75–1.28)	0.890
		C/C	38	58	0.61(0.39–0.95)	**0.028**	0.62(0.39–0.98)	**0.042**
	Dominant	G/G	289	269	1		1	
		C/C, G/C	248	260	0.89(0.70–1.13)	0.332	0.90(0.70–1.16)	0.417
	Recessive	G/G, G/C	499	471	1		1	
		C/C	38	58	0.62(0.40–0.95)	**0.028**	0.62(0.40–0.98)	**0.039**
	Additive	–	–	–	0.85(0.71–1.02)	0.087	0.86(0.71–1.04)	0.126
	Allele	G	788	740	1		1	
		C	286	318	0.85(0.70–1.02)	0.079	0.85(0.70–1.04)	0.118

^a^*p* values were calculated by logistic regression analysis

^b^*p* values were calculated by logistic regression analysis, after adjusting for age, gender, and BMI

The values of p and OR (95%CI), along with the statistical significance, are highlighted in bold

*SNV* Single nucleotide variation, *OR* Odds Ratio, *95%CI* 95% confidence interval

### Stratified analysis of rs15783G > A, rs1800517G > A, rs11960G > A, and rs5516C > G in the case–control study based on smoking status

COPD is a complex disease that is likely influenced by environmental factors, multiple genes, and gene-by-smoking/environmental interactions [12, 13]. To investigate whether the association between these four variants and COPD risk differed between smokers and non-smokers, we conducted a subgroup analysis (Table 4).

**Table 4:** Analysis of genotypes of all SNVs among non-smoker and smoker group

SNV ID	Model	Genotype	Non-smoking				Smoking			
			Case	Control	OR(95%CI)	*p*	Case	Control	OR(95%CI)	*p*
*ANO3/MUC15* rs15783	Codominant	A/A	158	171	1		45	25	1	
		A/G	203	206	1.01(0.74–1.37)	0.948	59	31	1.12(0.55–2.26)	0.754
		G/G	56	88	**0.63(0.42–0.96)**	**0.032**	20	12	0.90(0.36–2.25)	0.817
	Dominant	A/A	158	171	1		45	25	1	
		G/G, A/G	259	294	0.90(0.67–1.19)	0.450	79	43	1.05(0.55–2.03)	0.878
	Recessive	A/A, A/G	361	377	1		104	56	1	
		G/G	56	88	**0.63(0.43–0.92)**	**0.017**	20	12	0.84(0.37–1.94)	0.689
	Additive	–	–	–	0.84(0.68–1.02)	0.075	–	–	0.98(0.62–1.53)	0.914
	Allele	A	519	548	1		149	81	1	
		G	315	382	0.83(0.68–1.02)	0.071	99	55	0.98(0.62–1.54)	0.913
*COL4A4* rs1800517	Codominant	A/A	125	115	1		32	22	1	
		A/G	197	226	0.76(0.55–1.06)	0.110	58	32	1.16(0.56–2.46)	0.691
		G/G	95	124	**0.64(0.44–0.95)**	**0.026**	33	14	1.86(0.76–4.56)	0.174
	Dominant	A/A	125	115	1		32	22	1	
		G/G, A/G	292	350	**0.72(0.53–0.98)**	**0.039**	91	46	1.36(0.68–2.74)	0.388
	Recessive	A/A, A/G	322	341	1		90	54	1	
		G/G	95	124	0.77(0.55–1.06)	0.108	33	14	1.69(0.79–3.64)	0.178
	Additive	–	–	–	**0.80(0.66–0.97)**	**0.025**	–	–	1.35(0.87–2.10)	0.182
	Allele	A	447	456	1		122	76	1	
		G	387	474	**0.80(0.65–0.97)**	**0.023**	124	60	1.37(0.87–2.15)	0.169
RRBP1 rs11960	Codominant	A/A	128	165	1		42	14	1	
		A/G	212	215	1.21(0.88–1.65)	0.242	55	39	**0.41(0.19–0.89)**	**0.025**
		G/G	75	84	1.14(0.76–1.72)	0.521	27	15	0.39(0.15–1.01)	0.053
	Dominant	A/A	128	165	1		42	14	1	
		G/G, A/G	287	299	1.19(0.88–1.60)	0.254	82	54	**0.40(0.19–0.85)**	**0.018**
	Recessive	A/A, A/G	340	380	1		97	53	1	
		G/G	75	84	1.02(0.71–1.47)	0.908	27	15	0.71(0.33–1.53)	0.385
	Additive	–	–	–	1.09(0.89–1.33)	0.407	–	–	**0.62(0.39–0.98)**	**0.041**
	Allele	A	468	545	1		139	67	1	
		G	362	383	1.09(0.89–1.33)	0.406	109	69	**0.62(0.39–0.98)**	**0.039**
*KLK1* rs5516	Codominant	G/G	213	240	1		76	29	1	
		G/C	172	170	1.18(0.88–1.59)	0.267	38	32	**0.39(0.19–0.77)**	**0.007**
		C/C	29	52	0.64(0.38–1.08)	0.096	9	6	0.51(0.16–1.70)	0.276
	Dominant	G/G	213	240	1		76	29	1	
		C/C, G/C	201	222	1.06(0.80–1.40)	0.697	47	38	**0.41(0.21–0.78)**	**0.007**
	Recessive	G/G, G/C	385	410	1		114	61	1	
		C/C	29	52	**0.60(0.36–0.99)**	**0.046**	9	6	0.78(0.25–2.46)	0.671
	Additive	–	–	–	0.94(0.76–1.16)	0.557	–	–	**0.56(0.34–0.93)**	**0.024**
	Allele	G	598	650	1		190	90	1	
		C	230	274	0.94(0.75–1.16)	0.549	56	44	**0.55(0.33–0.91)**	**0.020**

*p* values were calculated by logistic regression analysis, after adjusting for age, gender, and BMI

The values of *p* and OR (95% CI), along with the statistical significance, are highlighted in bold

*SNV* Single nucleotide variation, *OR* Odds Ratio, *95%CI* 95% confidence interval

We found that *ANO3/MUC15* rs15783 and *COL4A4* rs1800517 polymorphisms were only related to an altered risk of COPD in non-smokers (*p* < 0.05). Individuals with the *ANO3/MUC15* rs15783 G/G genotype had a reduced risk in the codominant (OR = 0.63, *p* = 0.032) and recessive (OR = 0.63, *p* = 0.017) models. Another *COL4A4* rs1800517G > A polymorphism also played a protective role in non-smokers, and carriers of the minor allele (G) had a lower risk for COPD, based on the results for the allele (OR = 0.80, *p* = 0.023) and additive (OR = 0.80, *p* = 0.025) models. Additionally, the *COL4A4* rs1800517 G/G genotype was also associated with a decreased risk of COPD in the codominant (OR = 0.64, *p* = 0.026) and dominant (OR = 0.72, *p* = 0.039) models.

The *RRBP1* rs11960G > A played a protective role in smokers (*p* < 0.05). The *RRBP1* rs11960 A/G genotype was associated with a decreased risk of COPD, based on the results obtained with the codominant (OR = 0.41, *p* = 0.025) and dominant (OR = 0.40, *p* = 0.018) models. The *RRBP1* rs11960G > A SNV fitted the additive model, and a significantly decreased risk of COPD was observed in the presence of a “G” allele (OR = 0.62, *p* = 0.041).

We observed that the *KLK1* rs5516C > G polymorphism influenced COPD risk in both smokers and non-smokers in different genetic models. The results of the stratified analyses for the recessive model (OR = 0.60, *p* = 0.046) indicated that the *KLK1* rs5516 C/C genotype was associated with a decreased risk of COPD in non-smoking participants. However, we also found that the *KLK1* rs5516 polymorphism was strongly related to COPD risk in smoking participants, while carriers of the G/C genotype were at lower risk for COPD, based on the results obtained with the codominant (OR = 0.39, *p* = 0.007) and dominant (OR = 0.41, *p* = 0.007) models. The *KLK1* rs5516C > G SNV fitted the additive model, and was observed to be associated with a significantly decreased risk of COPD in the presence of a "C" allele (OR = 0.56, *p* = 0.024).

### Stratified analysis of rs15783G > A, rs1800517G > A, rs11960G > A, and rs5516C > G based on COPD severity in the case–control study

Using a logistic regression model, we investigated the association of *ANO3/MUC15*, *COL4A4*, *RRBP1*, and *KLK1* genotype distributions and disease severity based on lung function, using a cutoff limit of FEV1 = 50% predicted. As shown in Table 5, no significant correlation was found between the genotype distribution of *ANO3/MUC15* rs15783G > A, *COL4A4* rs1800517G > A, *RRBP1* rs11960G > A, and *KLK1* rs5516C > G polymorphisms and airflow limitation severity (*p* > 0.05).

**Table 5:** Analysis of genotypes of all SNVs at different FEV1 predicted values in COPD patients

SNV ID	Model	Genotype	FEV1 predicted, n		OR(95%CI)	*p*
			≥ 50%	< 50%		
*ANO3/MUC15* rs15783	Codominant	A/A	167	36	1	
		A/G	210	52	0.88(0.55–1.41)	0.584
		G/G	65	11	1.29(0.62–2.70)	0.497
	Dominant	A/A	167	36	1	
		G/G, A/G	275	63	0.95(0.60–1.49)	0.819
	Recessive	A/A, A/G	377	88	1	
		G/G	65	11	1.39(0.71–2.76)	0.340
	Additive	–	–	–	1.06(0.76–1.46)	0.743
	Allele	A	544	124	1	
		G	340	74	1.05(0.77–1.45)	0.746
*COL4A4* rs1800517	Codominant	A/A	125	32	1	
		A/G	213	42	1.33(0.80–2.22)	0.276
		G/G	103	25	1.08(0.60–1.95)	0.792
	Dominant	A/A	125	32	1	
		G/G, A/G	316	67	1.24(0.77–1.98)	0.376
	Recessive	A/A, A/G	338	74	1	
		G/G	103	25	0.91(0.55–1.51)	0.720
	Additive	–	–	–	1.06(0.78–1.43)	0.731
	Allele	A	463	106	1	
		G	419	92	1.06(0.78–1.44)	0.725
*RRBP1* rs11960	Codominant	A/A	134	36	1	
		A/G	221	46	1.33(0.81–2.17)	0.259
		G/G	85	17	1.40(0.74–2.65)	0.306
	Dominant	A/A	134	36	1	
		G/G, A/G	306	63	1.346(0.85–2.14)	0.207
	Recessive	A/A, A/G	355	82	1	
		G/G	85	17	1.18(0.66–2.10)	0.570
	Additive	–	–	–	1.21(0.88–1.66)	0.248
	Allele	A	489	118	1	
		G	391	80	1.20(0.88–1.65)	0.250
*KLK1* rs5516	Codominant	G/G	231	58	1	
		G/C	176	34	1.30(0.82–2.08)	0.278
		C/C	31	7	1.19(0.49–2.85)	0.702
	Dominant	G/G	231	58	1	
		C/C, G/C	207	41	1.28(0.82–2.00)	0.279
	Recessive	G/G, G/C	407	92	1	
		C/C	31	7	1.07(0.45–2.52)	0.877
	Additive	–	–	–	1.18(0.83–1.70)	0.357
	Allele	G	638	150	1	
		C	238	48	1.18(0.83–1.70)	0.356

*P* values were calculated by logistic regression analysis, after adjusting for age, gender, and BMI

*FEV1* Forced expiratory volume in 1 s, *SNV* Single nucleotide variation, *OR* Odds Ratio, *95%CI* 95% confidence interval

## Discussion

COPD is a heterogeneous and complex disease, and the genetic composition of an individual could significantly determine susceptibility [3–5]. We first screened four mutations, namely, *ANO3/MUC15* rs15783, *COL4A4* rs1800517, *RRBP1* rs11960, and *KLK1* rs5516, which were identified to be associated with COPD via a WES analysis of a cluster of three COPD families. The results of association analysis for these four SNVs indicated that the *ANO3/MUC15* rs15783 and *KLK1* rs5516 polymorphisms were associated with COPD susceptibility in the entire population. Further, upon performing stratified analysis, we found that the *ANO3/MUC15*, *KLK1*, *COL4A4*, and *RRBP1* polymorphisms were closely associated with a reduced risk of COPD in individuals with different smoking statuses, but were not related to disease severity.

The *ANO3/MUC15* rs15783, *COL4A4* rs1800517, *RRBP1* rs11960, and *KLK1* rs5516 are non-synonymous variants (via NCBI database: https://www.ncbi.nlm.nih.gov/). Three of the 4 SNVs (*COL4A4* rs1800517, *RRBP1* rs11960, and *KLK1* rs5516) are exonic SNPs and correspond to a missense substitution. Only SNV rs15783 occurs in the overlapping genes *ANO3* and *MUC15*; it occurs as an intronic SNP in *ANO3* (intron 13 of NM_031418.2 transcript) and exonic SNP in *MUC15*, and corresponds to a missense substitution (Thr > Ile) at position 229 in the *MUC15* isoform b [14]. The enrichment analyses of the Gene Ontology (GO) database (http://www.geneontology.org) and Kyoto Encyclopedia of Genes and Genomes (KEGG) pathway (https://www.genome.jp/kegg) indicated that the four genes did not occur in the same KEGG metabolic pathway. In addition, protein interaction network analysis using the STRING database (https://string-db.org/) showed that these proteins did not interact with each other in any manner.

Prolonged exposure to CS might increase the total burden attributable to environmental factors such as genetic predisposition, which might increase the risk of COPD sufficiently and cause COPD [12, 13], especially in individuals with high levels of susceptibility. In the present study, we first demonstrated that the *ANO3/MUC15* rs15783 and *COL4A4* rs1800517 SNVs were associated with a reduced risk of COPD in non-smokers. Notably, smokers did not have a significantly altered risk of COPD, regardless of the genetic model, compared to non-smokers. This suggests that the G/G genotype of *ANO3/MUC15* rs15783 and allele G of *COL4A4* rs1800517 might exhibit only a weak protective effect against COPD in non-smokers; this protective relationship would be reversed if non-smokers carrying protective mutations at these two loci were affected by CS. Here, the *RRBP1* rs11960 polymorphism was also first shown to be associated with COPD susceptibility in smokers; an individual with allele G could have a significantly reduced risk of COPD. Interestingly, we observed that this protective effect of *RRBP1* rs11960 could be observed only in those carrying heterozygous mutations. In addition, we found that the risk of COPD was decreased in smokers with the *KLK1* rs5516 G/C genotype and non-smokers with the rs5516 C/C variant, though individuals with different smoking statuses were affected by homozygous and heterozygous mutations.

COPD manifests as persistent airflow obstruction. Although mucus and other epithelial secretions of the airway play a critical role in protecting the lung during acute injury, impaired mucus clearance after chronic mucus hyperproduction causes airway obstruction, infection, and inflammation, which contribute to morbidity in common pulmonary disorders, including chronic obstructive pulmonary disease and asthma [15]. *ANO3* (also known as *TMEM16C*) belongs to the *TMEM16* family encoding predicted membrane proteins that are involved in the functioning of intracellular calcium-activated chloride channels (CaCCs). These proteins perform many important functions in cell physiology, including the facilitation of fluid secretion from acinar cells of secretory glands, regulation of neuronal excitability, and regulation of smooth muscle contraction [16]. Although limited information is available regarding the function of TMEM16C in COPD pathology, another anoctamin (TMEM16A) has been shown to be involved in CaCC-based mechanisms resulting in excessive mucus secretion and airway smooth muscle contraction during inflammatory airway disease [17]. The process of GO annotation of genes related to *TMEM16A* and *TMEM16C* includes genes that facilitate intracellular CaCC activity, suggesting that *TMEM16C* might also be involved in the pathophysiological mechanism of occurrence of COPD, via the regulation of mucus secretion or airway smooth muscle contraction. However, studies on *ANO3* have mainly focused on genetic dystonia [18]. *MUC15* (Mucin 15, cell surface associated) belongs to the family of mucin genes, which encode large epithelial glycoproteins that are major constituents of the mucus that covers the surfaces of epithelial tissues and provides a physical barrier that protects the underlying epithelium [19]. MUC15 is a highly glycosylated protein exhibiting the structural features observed in other inline membrane mucins [20]. Therefore, we hypothesized that MUC15 might play a key role in the occurrence and development of COPD. However, studies on the correlation between MUC15 and human disease mainly focus on malignant tumors [21], and its correlation with COPD is still poorly understood. We showed that *ANO3/MUC15* rs15783 might act as a protective factor against COPD. It should be noted that both *ANO3* and *MUC15*, which are affected by the SNV rs15783, may be related to COPD; thus, *ANO3/MUC15* rs15783 SNV is highly likely to alter the risk of COPD.

*COL4A4* encodes one of the six subunits of type IV collagen, the major structural component of basement membranes that majorly contribute to the strength of the blood-gas barrier (BGB) [22]. The BGB (also known as the alveolar-capillary barrier) is the key functional element of the lung, and serves as the site of oxygen and carbon dioxide exchange between distal airspaces and the pulmonary vasculature [23]. A recent animal (mouse and chick) study found that type IV collagen plays a key role during alveolar morphogenesis and is critical for the proper formation of the BGB and the process of septation [22]. COPD is characterized by persistent respiratory symptoms and airflow limitations caused by airway and/or alveolar abnormalities that are influenced by a host of factors, including abnormal lung development [1]. An impaired microvascular barrier also has been observed in COPD patients [24, 25]. Meanwhile, it has been reported that type IV collagen is important for maintaining the integrity of the vascular basement membrane [26]. The important role of type IV collagen in the alveolar sphere and pulmonary vascular basement membrane structure suggests that a mutation in *COL4A4* might contribute to COPD occurrence. Although COL4A4 is widely expressed in both kidney (RPKM 5.3) and lung (RPKM 4.3) tissues (via NCBI database: https://www.ncbi.nlm.nih.gov/), previous studies have mainly focused on COL4A4 expression in the glomerular basement membrane [27, 28]. The specific role of COL4A4 in COPD development remains unknown. Our study has shown for the first time that *COL4A4* rs1800517 allele G may act as a protective factor against COPD. We speculated that the *COL4A4* rs1800517 SNV might prevent the occurrence of COPD as it affects basement membrane components in the lung tissue.

Endoplasmic reticulum stress (ERS) can be attributed to the improper folding or conformation (misfolded protein) of proteins, which can interfere with the normal physiological functions of the cell [29]. The unfolded protein response (UPR) is a mechanism by which cells control ER protein homeostasis [30]. RRBP1 was originally identified as a ribosome-binding protein located on the rough endoplasmic reticulum, and was majorly involved in regulating the secretion of intracellular proteins and alleviation of ERS [31–33]. ERS can be strongly induced by cigarette smoke extracts [34]. Several studies have indicated that the inhibition of ERS can largely ameliorate CS-induced airway inflammation and emphysema through the suppression of inflammation, apoptosis, and oxidative stress, via the blocking of ERS [34–37]. ERS has been implicated in COPD [38], while RRBP1 is associated with the regulation of UPR; however, the role of RRBP1 in COPD development is unclear. Here, the *RRBP1* rs11960 polymorphism was first shown to be a protective factor in smokers. Therefore, we speculated that RRBP1 might potentially exhibit protective effects against COPD by inhibiting CS-induced ERS.

A characteristic pathologic feature of COPD is a protease-antiprotease imbalance [39]. *KLK1* is one of the important regulatory genes contributing to a protease-antiprotease imbalance, and is essential for the pathogenesis of COPD. Previous studies have shown that the uncontrolled activity of KLK1 can lead to the direct proteolytic cleavage of the pro-epidermal growth factor, release of mature epidermal growth factor, and subsequent activation of epidermal growth factor receptor (EGFR), thereby resulting in metaplasia and excessive mucus secretion in individuals with airway diseases [40]. In addition, EGFR activation was also related to the pathologic phenotypes of epithelial cells in smokers [41]. Upon exposure to the reactive oxygen species derived from cigarette smoke or endogenous sources, the up-regulation of KLK1 activity leads to the degradation of the hyaluronic acid (HA) occurring on airway epithelial cells [42], subsequently weakening the limiting effect of the proteolytic activity of HA [43]. The *KLK1* rs5516 polymorphism influences susceptibility of multiple diseases and conditions, including aortic aneurysm [44], thoracic aortic dissection [45], SLE with nephritis [46], and essential hypertension [47]. To date, this is the first study to demonstrate that *KLK1* rs5516 SNV might be associated with COPD susceptibility.

We used a combination of whole-exome and targeted sequencing to search for novel genetic characteristics of COPD in the Chinese Uighur population. To our knowledge, we are the first to report the association between the four SNVs and COPD risk. However, several limitations are associated with our study. First, our population verification process is based on the ethnic characteristics of individuals from Kashi, i.e., the Uyghur population. Therefore, the results are not representative of other ethnic groups. Second, the sample size of the subgroup of smokers was small; hence, our findings need to be confirmed in studies with a larger sample size of smokers. In addition, although polymorphisms of four genes identified in this study might be associated with lower prevalence of COPD, the specific role of these genes in COPD pathogenesis is unclear. Additional investigations, including multi-omics approaches and functional studies are needed, to further determine disease causality and biological mechanisms of action of these genes.

## Conclusion

The present study identified four previously unreported mutations that were associated with COPD susceptibility in individuals with different smoking statuses. The *ANO3/MUC15* rs15783G > A and *COL4A4* rs1800517G > A polymorphisms had protective effects on non-smokers, while the *RRBP1* rs11960G > A polymorphism had protective effects on smokers. The *KLK1* rs5516C > G polymorphism was associated with a decreased risk of COPD, irrespective of whether the individual was a smoker or non-smoker. Our findings provide new insights regarding the contribution of the genetic makeup of an individual to COPD pathogenesis in a Chinese Uyghur population.

## Supplementary Information

**Additional file 1**. The uncropped image details of the location of SNV rs5516.

**Additional file 2**. The uncropped image details of the location of SNV rs11960.

**Additional file 3**. The uncropped image details of the location of SNV rs15783.

**Additional file 4**. The uncropped image details of the location of SNV rs1800517.

## Data Availability

The datasets used and analyzed during the current study are available from the corresponding author on reasonable request.

## Abbreviations

COPD: Chronic obstructive pulmonary disease
*ANO3/MUC15*: Anoctamin 3/Mucin 15
*COL4A4*: Collagen type IV Alpha 4 chain
*KLK1*: Kallikrein 1
*RRBP1*: Ribosome binding protein 1
CS: Cigarette smoking
WES: Whole-exome sequencing
SNVs: Single nucleotide variants
GOLD: Global initiative for chronic obstructive lung disease
FEV1: Forced expiratory volume in 1 s
FVC: Forced vital capacity
BMI: Body mass index
SNP: Single nucleotide polymorphism
MAF: Minor allele frequency
HWE: Hardy–Weinberg equilibrium
QQ: Quantile–quantile
SD: Standard deviation
ORs: Odds ratios
CIs: Confidence intervals
CaCCs: Calcium-activated chloride channels
GO: Gene ontology
BGB: Blood-gas barrier
ERS: Endoplasmic reticulum stress
UPR: The unfolded protein response
EGFR: Epidermal growth factor receptor
NIV: Noninvasive ventilation
SABA: Short-acting beta2-agonist
SAMA: Short-acting muscarinic antagonist
LABA: Long-acting beta2-agonist
LAMA: Long-acting muscarinic antagonist
ICS: Inhaled corticosteroids
LTRA: Leukotriene receptor antagonist
