# Supplementary figures and images for: Analysis of the association of ANO3/MUC15, COL4A4, RRBP1, and KLK1 polymorphisms with COPD susceptibility in the Kashi population
Source: BMC Pulm Med. 2022 May 5;22:178. doi: 10.1186/s12890-022-01975-3 (PMC9074245; doi:10.1186/s12890-022-01975-3)

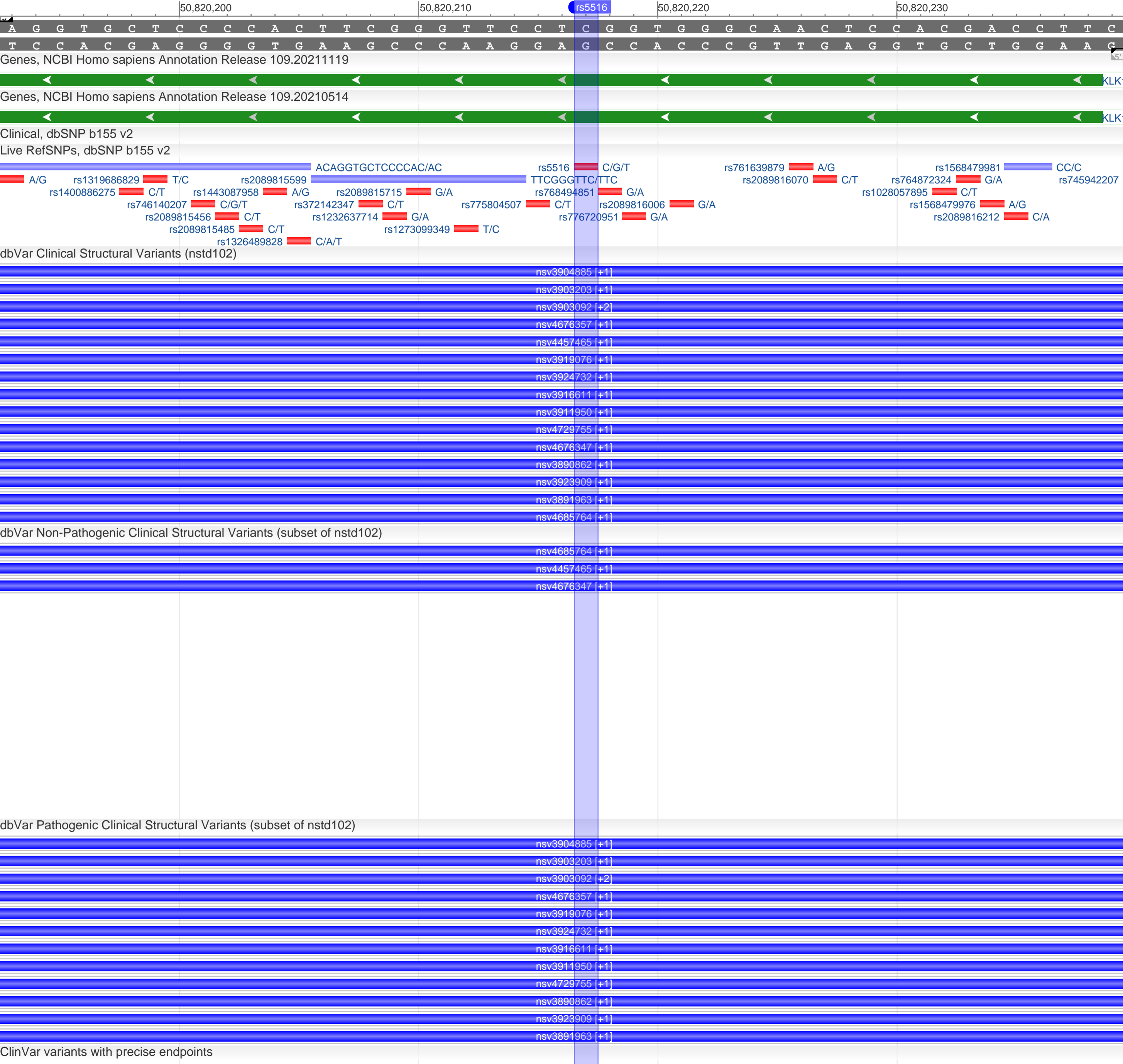

Supplement: Supplementary file 1 — Additional file 1. The uncropped image details of the location of SNV rs5516. [file 12890_2022_1975_MOESM1_ESM.pdf]

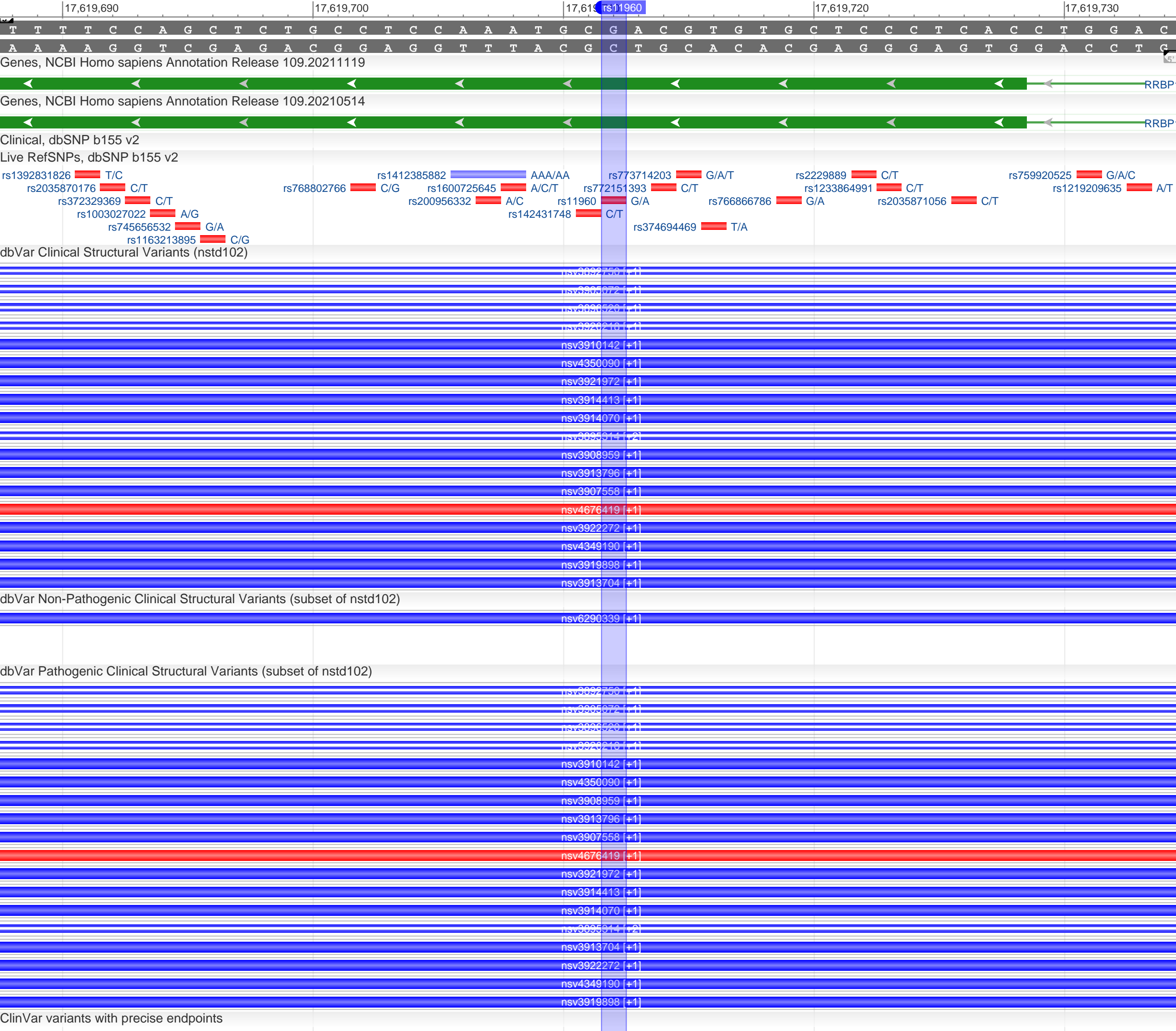

Supplement: Supplementary file 2 — Additional file 2. The uncropped image details of the location of SNV rs11960. [file 12890_2022_1975_MOESM2_ESM.pdf]

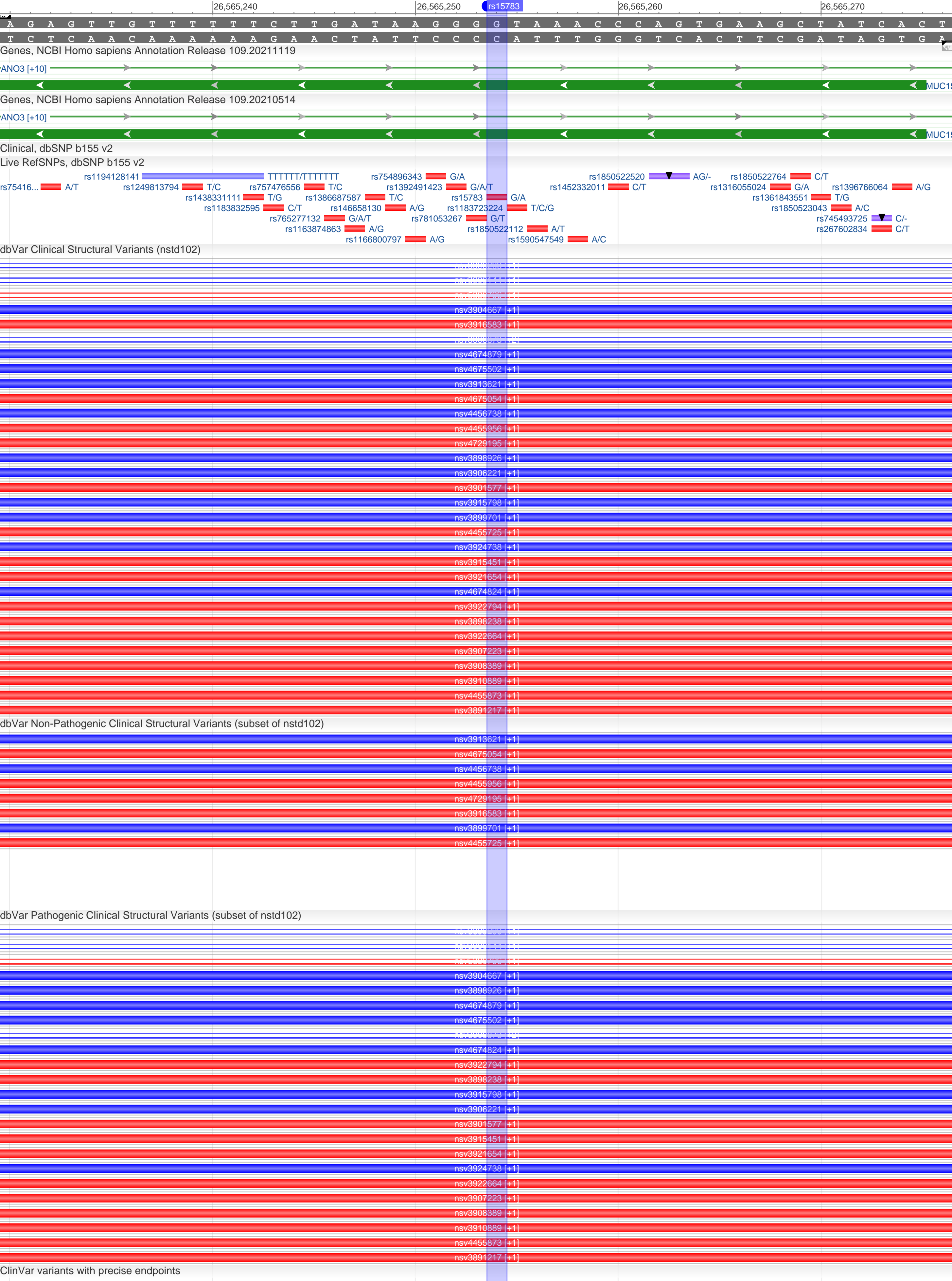

Supplement: Supplementary file 3 — Additional file 3. The uncropped image details of the location of SNV rs15783. [file 12890_2022_1975_MOESM3_ESM.pdf]

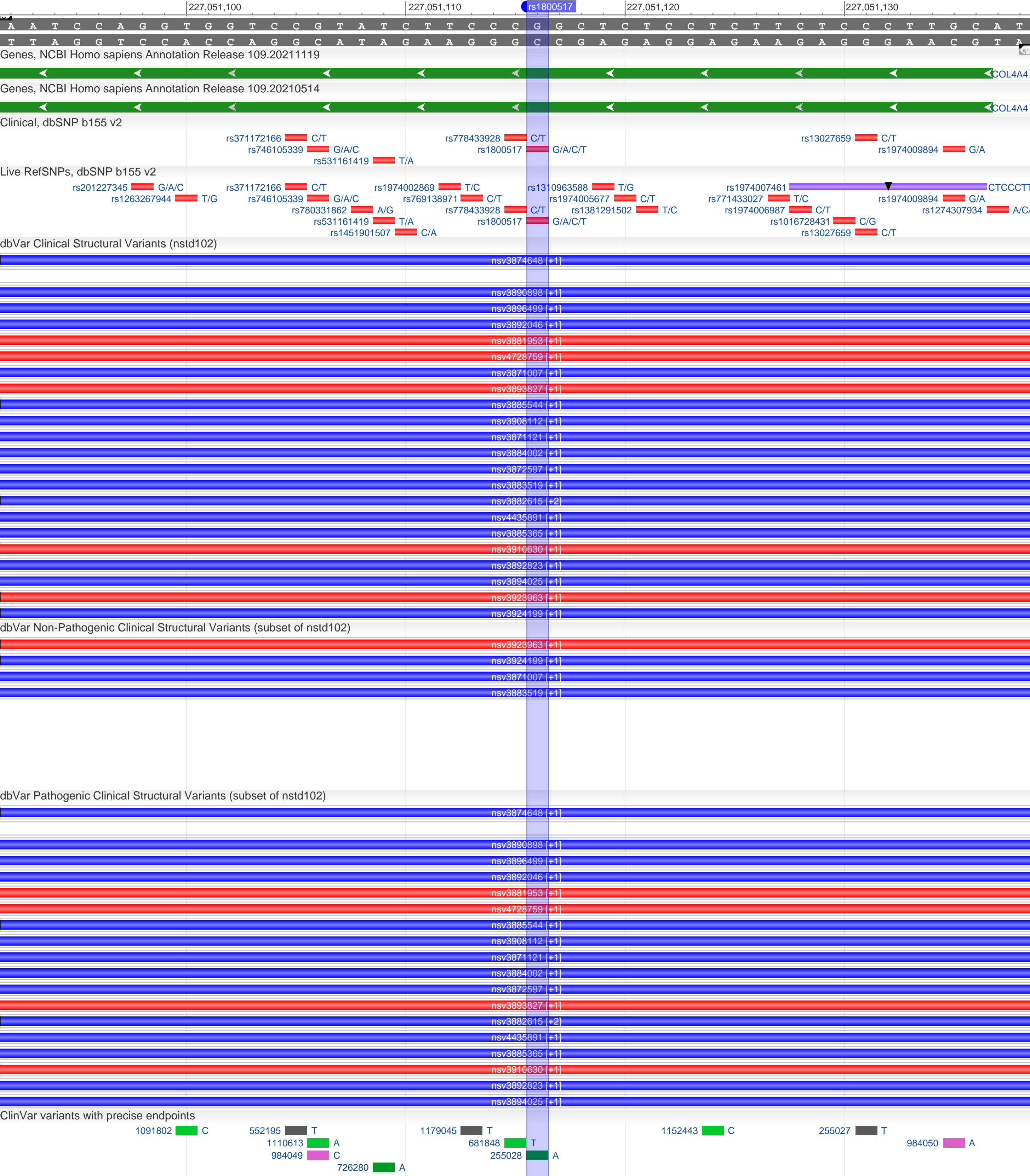

Supplement: Supplementary file 4 — Additional file 4. The uncropped image details of the location of SNV rs1800517. [file 12890_2022_1975_MOESM4_ESM.pdf]
